# The effect of introduction of routine immunization for rotavirus vaccine on paediatric admissions with diarrhoea and dehydration to Kenyan Hospitals: an interrupted time series study

**DOI:** 10.12688/wellcomeopenres.17420.1

**Published:** 2022-01-04

**Authors:** Daisy Chelangat, Lucas Malla, Reuben C. Langat, Samuel Akech, Clinical Information Network Author Group

**Affiliations:** 1 Health Services Unit, KEMRI-Centre of Geographic Medicine Research-Coast/ KEMRI-Wellcome Trust Research Programe, Nairobi, Kenya; 2 Department of Mathematics and Computer Science, University of Kabianga, Kericho, Kenya; 3 Infectious Disease Epidemiology, London School of Hygiene and Tropical Medicine, London, London, WC1E7HT, UK

**Keywords:** Diarrhea, dehydration, time series, rotavirus, vaccine, clinical information network, multiple imputation.

## Abstract

**Background:** Dehydration secondary to diarrhoea is a major cause of hospitalization and mortality in children aged less than five years. Most diarrhoea cases in childhood are caused by rotavirus, and routine introduction of rotavirus vaccine is expected to reduce the incidence and severity of dehydration secondary to diarrhoea in vaccinated infants. Previously, studies have examined changes in admissions with stools positive for rotavirus but this study reports on all admissions with dehydration secondary to diarrhoea regardless of stool rotavirus results. We aimed to assess the changes in all-cause severe diarrhoea and dehydration (DAD) admissions following the vaccine’s introduction.

**Methods:** We examined changes in admissions of all clinical cases of DAD before and after introduction of routine vaccination with rotavirus vaccine in July 2014 in Kenya. We use data from 13 public hospitals currently involved in a clinical network, the Clinical Information Network (CIN). Routinely collected data for children aged 2-36 months were examined. We used a segmented mixed effects model to assess changes in the burden of diarrhoea and dehydration after introduction of rotavirus vaccine. For sensitivity analysis, we examined trends for non-febrile admissions (surgical or burns).

**Results:** There were 17,708 patients classified as having both diarrhoea and dehydration. Average monthly admissions due to DAD for each hospital before vaccine introduction (July 2014) was 35 (standard deviation: ±22) and 17 (standard deviation: ±12) after vaccine introduction. Segmented mixed effects regression model showed there was a 33% (95% CI, 30% to 38%) decrease in DAD admissions immediately after the vaccine was introduced to the Kenya immunization program in July 2014. There was no change in admissions due to non-febrile admissions pre-and post-vaccine introduction.

**Conclusion:** The rotavirus vaccine, after introduction into the Kenya routine immunization program resulted in reduction of all-cause admissions of diarrhoea and dehydration in children to public hospitals.

## Introduction

Diarrhoea, passage of three or more loose stools in one day, causes dehydration when fluid loss exceeds intake or replacement, and rotavirus is a predominant infectious cause of diarrhoea in early childhood ( Kirk *et al*., 2017). Globally, approximately 1.7 billion diarrhoea cases are reported every year amongst children aged less than five years ( Heaton & Ciarlet, 2007). A survey in 2014 showed diarrhoea as the second leading cause of death in children aged less than five years in Kenya ( Mulatya & Mutuku, 2020) and is also a major cause of illness and death in children in other sub-Saharan African countries. Vaccination is one of the measures recommended by WHO for reducing severe diarrhoea and diarrheal deaths ( Kirk *et al*., 2017). Most severe diarrhoea cases from rotavirus occur in children aged between two to 36 months ( Fischer *et al*., 2002) and studies indicate that after 36 months of age, most survivors obtain natural immunity from rotavirus infection even if they have not been vaccinated.

Rotavirus vaccine, administered orally to children at six and ten weeks, was introduced as part of the routine Kenya Expanded Immunization Program (EPI) in July 2014 ( Wandera *et al*., 2017). Studies investigating the impact of the routine introduction of rotavirus vaccine in Kenya have shown a reduction in rotavirus positive diarrhoea cases, but these studies have been based on surveillance of rotavirus in stools of children admitted to sentinel hospitals and therefore miss the critical secondary effects of rotavirus vaccine in all-cause diarrhoea admissions ( Muendo *et al*., 2018; Otieno *et al*., 2020). In this study, we use routinely collected data to assess, using an interrupted time series design, the changes in all-cause severe diarrhoea admissions following the vaccine’s introduction. The study population comprises children admitted with diarrhoea and dehydration to public hospitals. 

## Methods

### Study area and setting

We use observational data collected from routine medical records from 13 public hospitals in Kenya participating in a Clinical Information Network (CIN). CIN is a collaboration to improve the collection and use of routine medical data to enhance the quality of care provided to admitted children through audit and feedback as previously described ( Ayieko *et al*., 2016; Gathara *et al*., 2017; Irimu *et al*., 2018a; Tuti *et al*., 2016). The collaboration is between the KEMRI-Wellcome Trust Research Program (KWTRP), Kenya’s Ministry of Health (MoH), the Kenya Pediatric Association, and participating county hospitals. Participation in the network by hospitals is voluntary but participating hospitals represent a wide geographical diversity of Kenya.

### Data capture in CIN hospitals

Standardized paediatric admission record (PAR) forms are used to capture the patient’s demographic and clinical details during admission, and discharge summary forms capture the patient’s discharge details, including diagnosis, and whether they are discharged alive or dead. The medical forms are filed together with laboratory reports and other notes documented by the clinician and form part of patients’ medical records. Participating hospitals have adopted these standardized forms as part of their routine medical records. Data is collected soon after the patient is discharged by abstracting data from the medical records into a dedicated database hosted in Research Electronic Data capture (REDCap), an open-source platform for capturing data ( Harris *et al*., 2009). Two categories of datasets are captured, minimum dataset and full dataset. Minimum datasets consist of information required for routine reporting to the ministry of health’s health management information system (HMIS) and consists of the patient’s demographic information, final diagnosis, and outcome (dead/alive). The full dataset consists of details on presenting history, admissions clinical assessment findings, admission treatments, details of investigations, and results of investigations. Minimum datasets are captured for children aged less than 30 days admitted to paediatric wards, surgical or burns admissions, and in randomized records in a few hospitals with high workload, and when the single data entry clerk is on leave for the high-volume hospitals ( Irimu *et al*., 2018b; Tuti *et al*., 2016). 

### Participants

The study population comprises children between the age of two and 36 months admitted with diarrhoea and dehydration from September 2013 to November 2019.

### Definitions of cases

Cases were identified as those with a discharge diagnosis of dehydration plus a history of diarrhoea or vomiting at admission (DAD-A) or presence of history of diarrhoea plus fulfilling criteria for signs of hypovolemic shock, severe dehydration or some dehydration (DAD-B). Severe dehydration is defined as presence of diarrhoea or vomiting with inability to drink or not alert plus either sunken eyes or return of skin pinch lasting two seconds or longer. A child is termed to be in a hypovolemic shock if they have all the following signs-a weak pulse volume, not alert, have cold hands, capillary refill time longer than three seconds plus sunken eyes and slow return of skin when pinched in the presence of diarrhoea or vomiting. Lastly, some dehydration is defined as the ability to drink with two or more of sunken eyes, or skin pinch taking 1- 2 seconds in children with diarrhoea or vomiting ( of Health, 2007).

### Statistical data analysis

**Figure 1.**
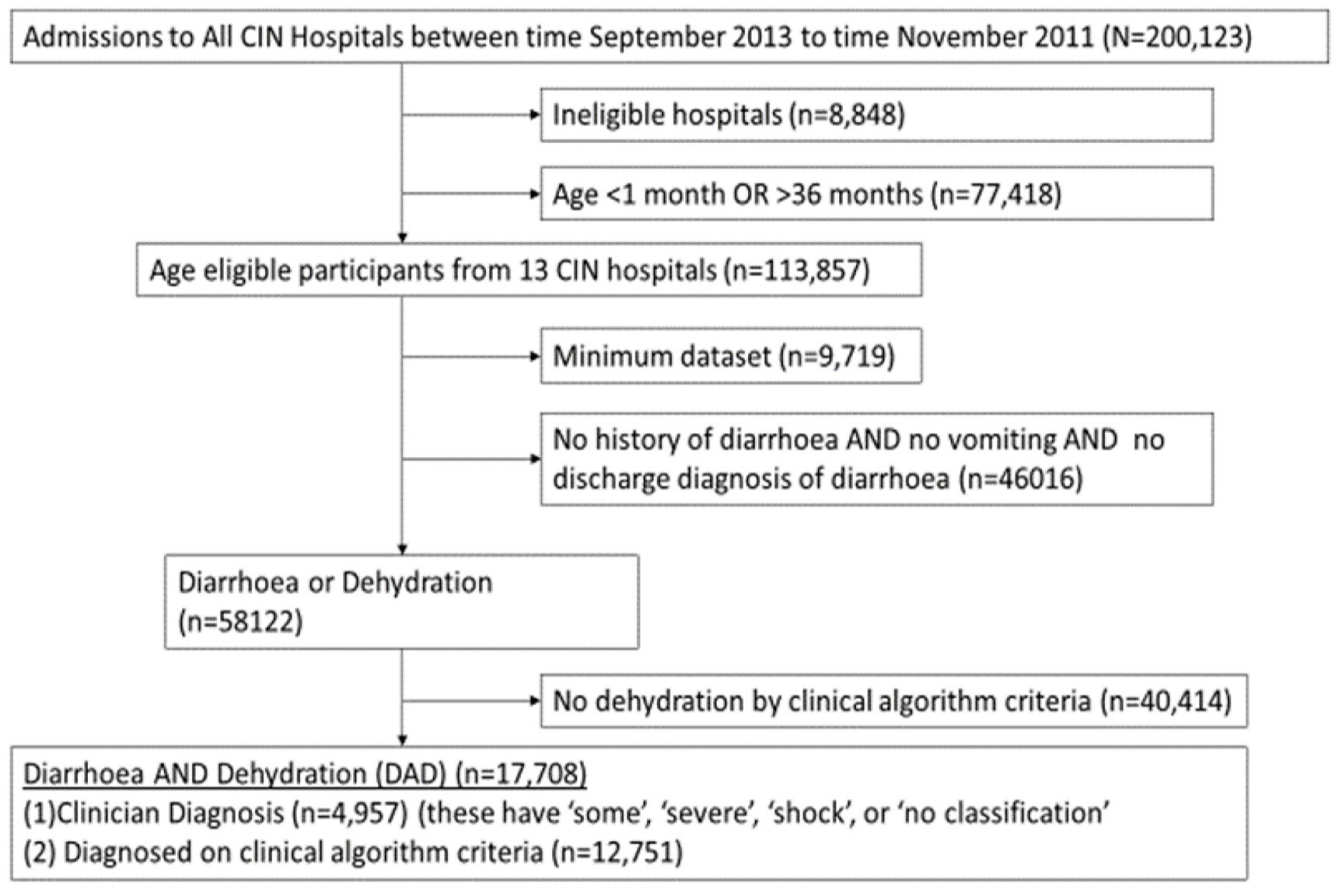
Patient inclusion criteria. Summary of how patients were selected for analysis.

As a first step, only hospitals which had data consistently from 2013 were selected and admissions restricted to only those patients whose ages were between 2 and 36 months ( Figure 1). We then selected those patients who either had a history of diarrhoea, vomiting, or a discharge diagnosis of diarrhoea or dehydration. Among the selected patients, there were those who were not indicated by the clinicians as having dehydration. We therefore used clinical signs recorded at admission to determine if children with history of diarrhoea met criteria for dehydration or shock as per the Kenya Basic Paediatric Protocols ( MOH, Kenya, 2016). Signs used included pulse rate, capillary refill time, temperature gradient, sunken eyes, skin pinch, alertness, and ability to drink. We first assessed these signs for completeness in documentation as missingness is an inherent analytical challenge in routine datasets ( Nicholls *et al*., 2017) as shown in Table 1. Secondly, we conducted multilevel multiple imputation to account for clustering of data within the hospitals. We did fifteen imputations and ten iterations under Missing At Random (MAR) assumption ( Schafer, 1999). Previous analysis of data from CIN hospitals have shown consistency with MAR assumption ( Gachau *et al*., 2019; Malla *et al*., 2019). On each of the imputed datasets, we proceeded to (i) sum the number of patients with diarrhoea and dehydration per month, both as classified by the clinicians and identified by the algorithms, and (ii) fit segmented mixed effects model with autoregressive covariance structure and with the counts following negative binomial distribution. The segmented mixed effects model examined whether there were changes in DAD cases immediately (step change) and whether there were any significant month to month changes (slope change) after July 2014. There were widespread hospital worker’s strikes between December 2016 to March 2017 and June 2017 to November 2017 and these strike periods were excluded in the analysis as there were very few to no admissions ( Irimu *et al*., 2018b). The modelling results across all the imputed datasets were pooled using Rubin rules ( Little & Rubin, 2019).

**Table 1.** Data completeness and distribution of DAD cases between complete and imputed datasets. summary of missing data per variable and completeness of features of interest before and after multiple imputation.

	Missing variable N=58,122	Proportion with DAD features in complete data N=58,122	Proportion with DAD features in imputed dataset N=58,122
**Female Sex**	0.7%(407)	44.5%	44.8%(26,055)
**Age**	0(0.0)	100%	100%
**Weak Pulse volume**	10.0% (5,854)	6.9% (4,016)	7.9% (4,574)
**Capillary refill time > 3 seconds**	15.3% (8,919)	10.4% (6,068)	12.7% (7,382)
**Temperature gradient**	17.2% (9,993)	5.6% (3,246)	7.0% (4,095)
**Delayed skin pinch**	9.8% (5,739)	23.7% (13,769)	26.7% (15,505)
**Sunken eyes**	9.5% (5,548)	19.6% (11y,403)	22.1% (12,866)
**AVPU score =V, P, or U**	6.1% (3,554)	7.3% (4,258)	7.9% (4,582)
**Inability to drink**	8.9% (5,190)	18.1% (10,523)	19.9%(11,571)

### Sensitivity analysis

In interrupted time series designs, it is critical to examine whether any changes observed would be attributable to the intervention under study and not any concurrent intervention(s) ( López Bernal, 2018). We therefore examined changes in admission patterns of surgical/burn patients for comparison with DAD admission patterns. Surgical/burns admissions were selected from the same hospitals as that of DAD and were also aged between two to 36 months. We then fitted a segmented mixed effects regression model with the outcome also following a negative binomial distribution. Significant impact of rotavirus vaccine would be inferred in case of any differences in step and slope changes in admission patterns between DAD and surgical/burn patients.

All the analyses were conducted using R version 4.0.0 ( *R: A Language and Environment for Statistical Computing*, n.d.)

### Ethics approval

Data used in this study is collected as part of routine medical records and individual patients’ consent is not obtained. The Ministry of Health (Kenya) and participating hospitals have given permission for CIN collaboration, which involves sharing routine data with the research group. Clinical Information Network study has been approved by the Kenya Medical Research Institute (KEMRI) Scientific and Ethical Review Unit (SERU), which has approved use CIN data for observational research without individual consenting (SERU #2465 and #3459).

## Results

### Patient selection

A total 17,708 children admitted to the 13 hospitals between September 2013 to November 2019 met eligibility criteria for diarrhoea and dehydration (DAD) ad shown in Figure 1. Imputation was done in admissions who fulfilled had diarrhoea or dehydration as shown in Figure 1 before final selection of the 17,708 admissions with DAD. The proportion of missing data for various variables for the 58,122 admissions with diarrhoea or dehydration (see Figures) and proportion with various characteristics in the complete cases and imputed datasets are shown in Table 1. A comparison of the proportion with features of interest before and after multiple imputation showed no difference in the imputed dataset.

### Participant’s summary statistics

We present results for the 17,708 patients classified as having both diarrhoea and dehydration (DAD). Average monthly admissions due to DAD for each hospital before vaccine introduction (July 2014) was 35 (standard deviation: ±22) and 17 (standard deviation: ±12) after vaccine introduction as summarized in Table 2. Hospital admissions per month in different hospitals ranged from 6 to 100.

**Table 2.** Participant’s summary statistics. Results of the exploratory analysis of the data.

	Overall DAD admissions (N=17,708)	Per hospital Before July 2014 N=3,429	DAD per hospital After July 2014 N=14,297
**Median Age in months, (interquartile range)**	13.9(8-18)	13.57 (8-18)	13.97(8-19)
**Mean Monthly DAD admissions per hospital** **(±standard deviation)**	19(±15)	35(±22)	17 (±12)
**Median monthly admissions per hospital** **(interquartile range)**	14 (9-23)	30 (17-45))	14 (9-21)
**Proportion of in-hospital deaths, n (%)**	2.5% (4497)	1.7% (584)	2.7% (3,910)

### Changes in diarrhoea and dehydration after introduction of rotavirus vaccine

**Figure 2.**
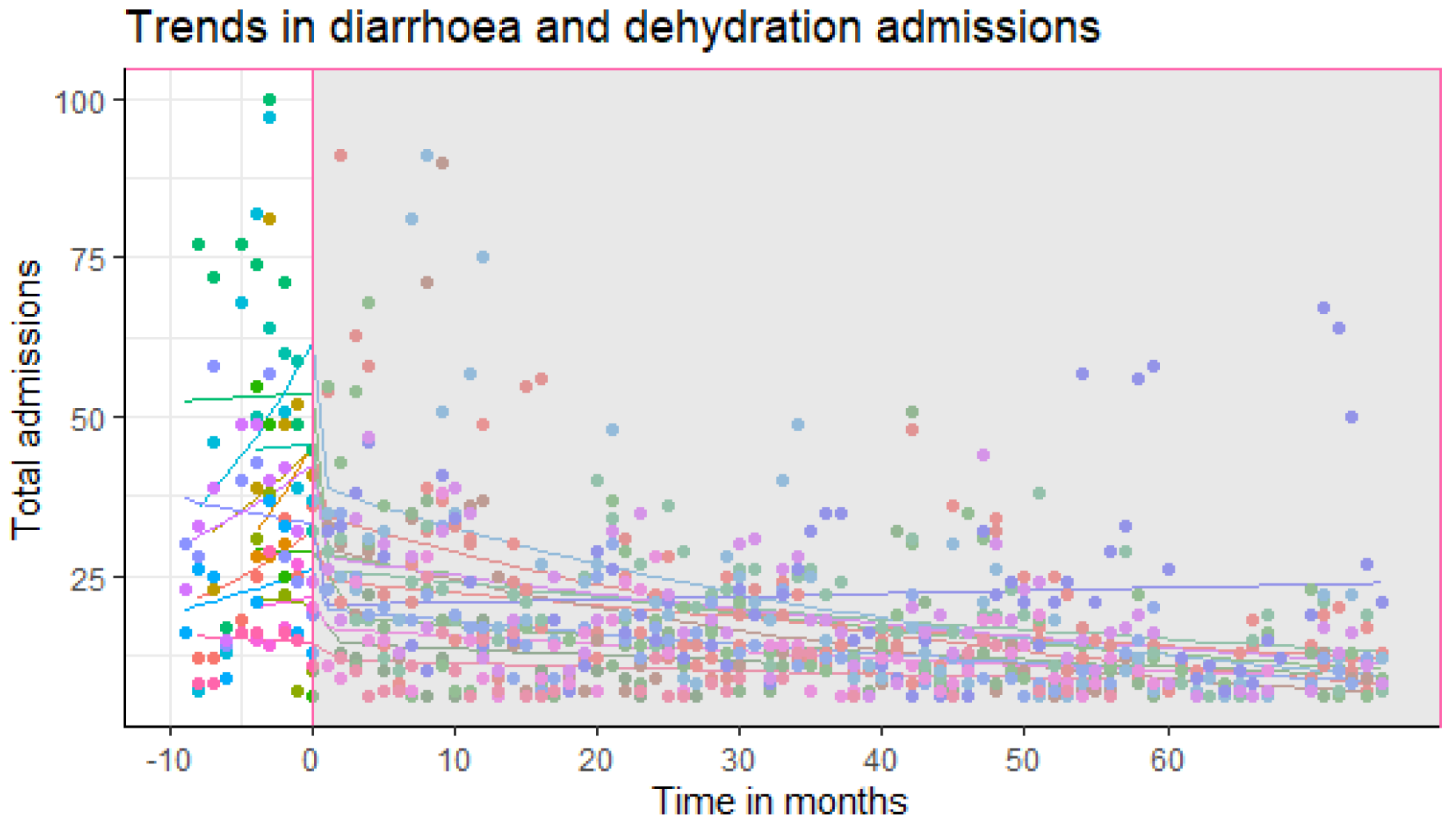
Trends in hospitalization due to diarrhoea and dehydration. Slope and level change in DAD hospitalizations over time.

There was a 33.33% (95% Confidence Interval (CI): 15% to 45%) decrease (step change) in DAD admissions immediately after the vaccine was introduced to the Kenya Immunization Program in July 2014. The preceding 3.00% (95% CI: -3% to 9%) month to month change in slope in hospital admissions due to all-cause diarrhoea and dehydration was not statistically significant as presented in Table 3 and Figure 2. 

**Table 3.** Interrupted time series analysis coefficients for diarrhoea and dehydration admissions. regression analysis results showing Change in both slope and level of hospitalization due to diarrhoea and dehydration after the introduction of rota virus vaccine in July 2014.

	Odds Ratios	95% confidence interval	P- value
**(Intercept)**	25.41	24.90 to 27.01	0
**Time**	1.02	0.97 to 1.09	0.50
**Level change**	0.67	0.55 to 0.85	0.00
**Slope change**	0.97	0.91 to 1.03	0.23

Note: Time - change in the slope of DAD admissions before July 2014; level change - change in admissions immediately after July 2014; slope change- change in the slope of admissions after July 2014

### Trends in surgical and burns admissions

**Figure 3.**
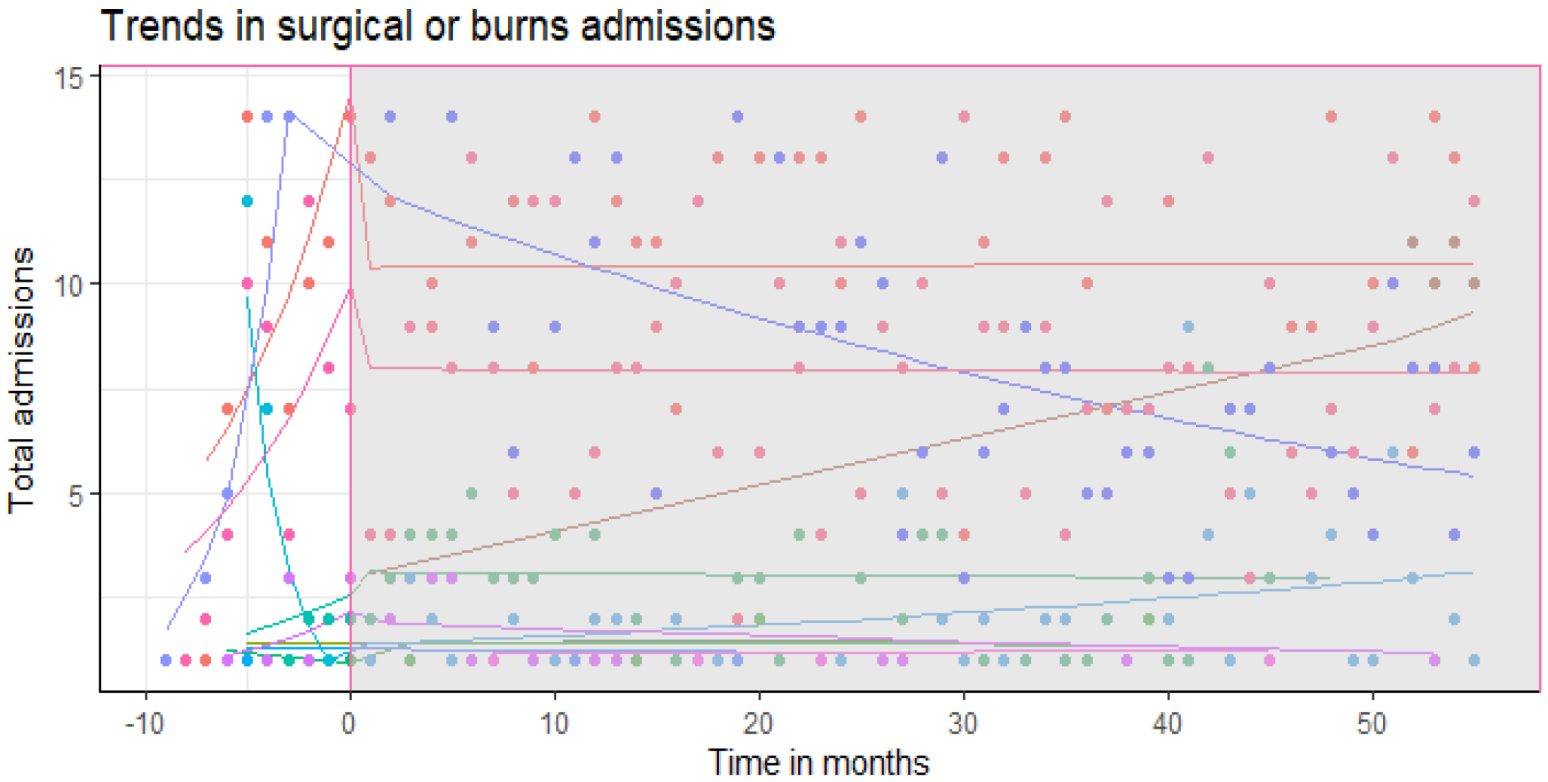
Trends in hospitalization due to diarrhoea and dehydration. Slope and level change in DAD hospitalizations over time.

We analysed 2,960 eligible admissions due to surgical or burns cases. The mean admissions of surgical or burns cases pre-intervention period was 41 patients (standard deviation ±12.72) and 36 patients (standard deviation ±8.16) post intervention. Our segmented negative binomial regression model showed no significant changes both in step and slope in hospitalization patterns due to burns ( Table 4 and Figure 3) post July 2014 when the rotavirus vaccine was introduced. Change in month to month admissions (slope change) was -6% (95% CI: -38% to 2%) while step change was -25% (95% CI: -4% to 42%)

**Table 4.** Interrupted Time Series regression coefficients showing change in admissions due to surgical or burns. Results of regression model accessing change in hospitalization due to surgical or burns after the introduction of rota virus vaccine in July 2014.

Parameters	Rate Rations	95% confidence interval	p-values
**Intercept**	3.39	0.62 to 3.94	0.15
**Time**	0.94	0.73 to 1.20	0.66
**Level change**	1.25	0.58 to 1.04	0.58
**Slope change**	1.06	0.98 to 1.38	0.65

Note: Time - change in the slope of burns admissions before July 2014; level change - change in admissions immediately after July 2014; slope change- change in the slope of admissions after July 2014

## Discussion

This study reveals an overall reduction in hospital admissions due to all-cause diarrhoea and dehydration following the introduction of the rotavirus vaccine for children most at risk of rotavirus diarrhoea (2 to 36 months). Despite introduction of the vaccine in 2014, there remains significant admissions of cases of diarrhoea with stools positive for rotavirus in Kenya ( Akech *et al*., 2018; Muendo *et al*., 2018; Nyaga *et al*., 2018). Analyses specific to rotavirus positive cases from stool samples, seeking to evaluate vaccine performance, have shown reduction in hospitalization ( Otieno *et al*., 2020; Wandera *et al*., 2017). Our study, which does not rely on rotavirus positive stool samples, further demonstrate benefit of introduction of rotavirus vaccine for reduction of cases of dehydration secondary to diarrhoea even in the absence of a stool test.

Pre-post analysis of the data showed a reduction in mean DAD hospitalization after the intervention. The fitted regression analysis model also showed an immediate reduction in all-cause DAD hospitalization following vaccination. This indicates an association between the change in children’s volumes admitted to hospital due to all-cause DAD and the period of vaccine introduction. During the same study period, we observed no change in admissions with surgical/burns cases that were used as controls. This result is consistent to a study published in 2019 conducted in Kilifi county, Kenya ( Otieno *et al*., 2020). In the study, a surveillance was carried out for hospitalized children under the age of five and stools were tested for rotavirus. Data was collected from 2010 to 2017 which showed a significant effect of the vaccine in reducing rotavirus positive hospitalizations in the age group.

 The results are also consistent with a recent study in Kenyan seeking to explore the prevalence of diarrhoea causing viruses in coastal Kenya before and after introduction of the rotavirus vaccine. Patients’ stool samples were screened for different types of viruses and they showed that rotavirus prevalence had reduced post the intervention period ( Wandera *et al*., 2017). Our findings are in line with the results of a recent systematic review involving 34 sub-Saharan countries who had introduced the vaccine into their routine immunization program where studies reporting rotavirus positive cases in children aged less than five years were included ( Godfrey *et al*., 2020). It was observed that there was a significant relationship with the reduction of rotavirus infection and use of the vaccine.

The main contribution of our study to the growing literature on the impact of rotavirus vaccine is that we use routine data collected from medical notes and demonstrate the impact of the vaccine in all-cause diarrhoea admissions. We show the value of routine hospital data to investigate impact of interventions, which could be valuable to supplement case control studies or surveys that often require significant resources to set up. Use of routinely collected data is cost effective, generalizable for severe cases with access to hospital care and they provide an attractive option for evaluation of effectiveness of interventions post implementation ( Ayieko *et al*., 2016; Irimu *et al*., 2018a; Tuti *et al*., 2016). 

Our results are unlikely to be biased due to several reasons; we limited our analysis to children aged less than three years, the age most at risk of severe diarrhoea from rotavirus inflection. Diagnostics for multiple imputation showed that our imputation model yielded plausible values as shown in Table 1 where there is no difference in the proportion of observations with various characteristics post imputation.

This study assumes that patients use of the health facilities where not affected by other external factors in the two periods. However, significantly low admissions were recorded during the strike periods from December 2016 to March 2017 and July to November 2017. These periods were excluded from our study. We use data from 13 hospitals spread from across the country and admissions are unlikely to have been affected by localized factors such as establishment of major competing health facility. The pre-intervention period was eleven months which is shorter when compared to the 54 months post-intervention period. However, this is not a threat to validity of the analytic approach as many studies have shown that a minimum of ten datapoints was sufficient to detect change due to an intervention ( López Bernal, 2018).

## Conclusion

The rotavirus vaccine, after introduction into the Kenya routine immunization program, has resulted in reduced all-cause admissions of diarrhoea and dehydration in children aged less than 36 months to public hospitals in Kenya. The study demonstrates the value of routine hospital data for monitoring impact of interventions.

## Data availability

### Underlying data

Harvard Dataverse: CIN paediatric admissions, https://doi.org/10.7910/DVN/C0CDP9 ( Chelangat *et al*., 2021).

Data for this report are under the primary jurisdiction of the Ministry of Health in Kenya and are not openly available. The data used are available upon request by submitting a formal request through the KWTRP Data Governance Committee via email: dgc@kemri-wellcome.org. The details of the data access guidelines can be found on the KEMRI Wellcome Trust data repository ( https://dataverse.harvard.edu/dataverse/kwtrp). Access can also be requested through Harvard Dataverse.

The data codebook (KWTRP_DATA_CODEBOOK_Daisy.docx) is available under the terms of the Creative Commons Attribution 4.0 International license (CC-BY 4.0).
