# Supplementary material for: Examining the impact of implementing routine rotavirus vaccination on the number of paediatric admissions due to diarrhoea and dehydration in Kenyan hospitals: A study using interrupted time series analysis
Source: Wellcome Open Res. 2025 Jan 8;7:2. Originally published 2022 Jan 4. [Version 2] doi: 10.12688/wellcomeopenres.17420.2 (PMC11782930; doi:10.12688/wellcomeopenres.17420.2)
Supplement: Peer review history

# Reviewer report: Reviewer response for version 2

**DOI:** 10.21956/wellcomeopenres.25455.r117738

**Published:** 2025-02-07

**Authors:** Flavia Kaduni Bawa

**Affiliations:** 1 Biochemistry, Cell and Molecular Biology, University Ghana, Legon, Accra, Ghana

**Abstract:** Can we say the immunisation program “contributed” to the reduction in admissions rather than “resulted in” since rotavirus is not the only cause of diarrhoea?

**Methodology:** It is unclear whether there was a mass vaccination of eligible children after the vaccine was introduced. At 9 months, most children would have taken most of their vaccinations requiring them to go to the vaccination centre less frequently. In this case, they would have missed the vaccination after completing their routine visits. Can you confirm whether these children were vaccinated in a mass vaccination? Otherwise, we cannot assume that children who came to the hospital after the vaccination program was rolled out were vaccinated. This could only be confirmed for those who turned 6 weeks after the vaccine introduction.

 Does the strike period stated coincide with the peak seasons of rotavirus infections and could this have affected your results? This has been stated in the limitation but not compared to peak seasons

**Results: **If you have data for just one year before the vaccine was introduced, could you have analyzed for one year after the vaccine was introduced instead such that they would be comparable? Also, is it possible to show the monthly distribution of cases between pre and post-vaccination to compare peak seasons and changing trends?

Is the work clearly and accurately presented and does it cite the current literature?

Yes

If applicable, is the statistical analysis and its interpretation appropriate?

I cannot comment. A qualified statistician is required.

Are all the source data underlying the results available to ensure full reproducibility?

Yes

Is the study design appropriate and is the work technically sound?

Yes

Are the conclusions drawn adequately supported by the results?

Partly

Are sufficient details of methods and analysis provided to allow replication by others?

Partly

Reviewer Expertise:

Infectious diseases, febrile illness

I confirm that I have read this submission and believe that I have an appropriate level of expertise to confirm that it is of an acceptable scientific standard, however I have significant reservations, as outlined above.

# Reviewer report: Reviewer response for version 2

**DOI:** 10.21956/wellcomeopenres.25455.r117024

**Published:** 2025-01-30

**Authors:** Mavuto Mukaka

**Affiliations:** 1 Mahidol Oxford Research Unit, Mahidol University, Bangkok, Thailand

The authors have adequately addressed my comments. I have no further comments.

Is the work clearly and accurately presented and does it cite the current literature?

Partly

If applicable, is the statistical analysis and its interpretation appropriate?

Partly

Are all the source data underlying the results available to ensure full reproducibility?

No

Is the study design appropriate and is the work technically sound?

Yes

Are the conclusions drawn adequately supported by the results?

Yes

Are sufficient details of methods and analysis provided to allow replication by others?

Partly

Reviewer Expertise:

Statistics, epidemiology, malaria

I confirm that I have read this submission and believe that I have an appropriate level of expertise to confirm that it is of an acceptable scientific standard.

# Reviewer report: Reviewer response for version 2

**DOI:** 10.21956/wellcomeopenres.25455.r117396

**Published:** 2025-01-20

**Authors:** Tayebeh Latifi

**Affiliations:** 1 Biochemistry, Emory University, Atlanta, Georgia, USA

While the rotavirus vaccine has reduced the number of rotavirus cases, it is important to recognize that rotavirus is not the only cause of diarrhea-associated deaths (DAD). Ignoring other potential agents introduces a bias in the study’s conclusions.

 As mentioned in the paper, many studies have already demonstrated a reduction in rotavirus cases and diarrhea following vaccine introduction. The authors need to clarify what is novel about their findings compared to existing literature.

**Comments**

**Introduction**

1. Mention the duration of the study (2013-2019).
2. Include the total number of patients (17,708) clearly.
3. In the background, correct the spelling of "diarrhea."

**Methods**

1. The paper states the study duration is 2014-2019, but this is not mentioned in the introduction. Ensure consistency.
2. Indicate how many of these cases received the rotavirus vaccine.

**Results**

1. Is there any significant difference between men and women before and after vaccination?
2. Specify what kind of rotavirus vaccine was used in those hospitals.
3. Figure 2: Note that the rotavirus vaccine and its type are not mentioned. Add more explanation to the figure legend.
4. What is the relevance of including surgical or burns cases? Why were these groups separated?

**For table 4:**

1. The legend does not explain the table very well. Add a more detailed presentation.
2. What is the logic behind making three different groups? What criteria were used?
3. Are the surgical and burns cases also from 2014-2019?

**Additional Suggestions for Reliability:** To enhance the reliability of the findings, compare the study’s data with rotavirus-positive stool samples collected during the study period. This would strengthen the conclusions and provide additional context for the vaccine’s impact.

Is the work clearly and accurately presented and does it cite the current literature?

Yes

If applicable, is the statistical analysis and its interpretation appropriate?

Partly

Are all the source data underlying the results available to ensure full reproducibility?

Partly

Is the study design appropriate and is the work technically sound?

No

Are the conclusions drawn adequately supported by the results?

No

Are sufficient details of methods and analysis provided to allow replication by others?

Partly

Reviewer Expertise:

Virology, Vaccine

I confirm that I have read this submission and believe that I have an appropriate level of expertise to state that I do not consider it to be of an acceptable scientific standard, for reasons outlined above.

# Reviewer report: Reviewer response for version 1

**DOI:** 10.21956/wellcomeopenres.19260.r53843

**Published:** 2023-02-13

**Authors:** Khitam Muhsen

**Affiliations:** 1 Department of Epidemiology and Preventive Medicine, School of Public Health, Sackler Faculty of Medicine, Tel Aviv University, Tel Aviv, Israel

The authors assessed the change in all-cause diarrhea and dehydration (DAD) hospital admissions after introducing universal rotavirus vaccination in Kenya (July 2014), using multicenter data of hospitalizations between September 2013 and November 2019.

 The topic of this manuscript is of interest. Nonetheless, some concerns need to be addressed.

 The authors used interrupted time-series analysis, but a limitation of this analysis is that there were very few data points before the introduction of rotavirus vaccination (September 2013 to July 2014).

 It is important to describe the observed data (monthly or weekly number [and rates if possible] of DAD hospitalizations.

 It is likely to take time to build up vaccinated birth cohorts in the community, accordingly is it possible to assume that there is a “transition period”, and not only “before and after periods”?

 Can the authors provide information on rotavirus vaccination coverage in Kenya over time?

 The changes in DAD before and after introducing universal rotavirus vaccination might vary with age groups since the vaccine is given to infants up to 32 weeks of age. Thus in the early period, the reduction might be of greater magnitude in infants than in toddlers. Therefore it is important to explore the change separately for different age groups (e.g. 0-11, 12-23, and 24-36 months).

 Additional comments

 Introduction – please provide up-to-date estimates (and references) of diarrheal disease burden.

 Tables/ figures: there are typo errors. Legends should be added to explain the tables/abbreviations, and statistical analysis.

 Table 2: instead of “mean monthly admissions per hospital” please present the median and interquartile range.

 Table 3, under P value, instead of 0.00, please use <0.001.

 In table 3 the authors presented odds ratios while in table 4 rate ratios. Please explain which models were used in each analysis.

 Table 4: please correct “ration” to ratios

 Figure 3: there is a discrepancy in the title: above the figure, it is written “trends in surgical or burns admissions”, while the title underneath the figure is” Figure 3. Trends in hospitalization due to diarrhoea and dehydration. Slope and level change in DAD hospitalizations over time”. Please check. In any case, this figure can be moved to supplementary material.

 In figures 2 and 3, please explain what the y-axis stands for rate or absolute numbers, as well as what the different colors of the dots represent

Is the work clearly and accurately presented and does it cite the current literature?

Partly

If applicable, is the statistical analysis and its interpretation appropriate?

Partly

Are all the source data underlying the results available to ensure full reproducibility?

Partly

Is the study design appropriate and is the work technically sound?

Partly

Are the conclusions drawn adequately supported by the results?

Partly

Are sufficient details of methods and analysis provided to allow replication by others?

Partly

Reviewer Expertise:

Epidemiology of infectious diseases and vaccines, rotavirus vaccination impact

I confirm that I have read this submission and believe that I have an appropriate level of expertise to confirm that it is of an acceptable scientific standard, however I have significant reservations, as outlined above.

# Response

**Published:** 2024-09-24

**Authors:** Daisy Chelangat

**Affiliations:** KEMRI-wellcome Trust, Kenya

**Comment**: The authors used interrupted time-series analysis, but a limitation of this analysis is that there were very few data points before the introduction of rotavirus vaccination (September 2013 to July 2014).

**Response**: We agree that the time we had a few timepoints before the introduction of the vaccine. However, from an analysis of literature it has been recommended for ITS studies to have at least eight time points pre-intervention(Ewusie et al., 2020). Our analysis had 10 timepoints which we believe provides a sufficient number.

**Comment**: It is important to describe the observed data (monthly or weekly number [and rates if possible] of DAD hospitalizations.

**Response**: We agree with this and a complete description of the CIN data including DAD data pre and post vaccine introduction have been added. “A total 200,123 paediatric patients were admited to CIN between September 2013 to November 2019 from 21 hospitals across Kenya. Admissions from eight  out of twenty one hospitals  were excluded from this study because they did not consistently have data from 2013 to 2019 (n = 8848). We further excluded 77,418 children who were not within our age range (1-36 months) in addition to 55735 who had only minimum data collected or no history of diahhrohea, no vomiting and no discharge diagnosis of diarrhoea (see Figure 1). A total of 58122 children were identified as having either dirrhoea or dehydration from which multiple imputation was applied to replace missing values. The proportion of missing data for various variables for the 58,122 admissions with diarrhea or dehydration and proportion with various characteristics in the complete cases and imputed datasets are shown in Table 1. A final sample of 17708 children met the eligibility criteria for diarrhea and dehydration (DAD). ….Average monthly admissions due to DAD for each hospital before vaccine introduction was 35 (standard deviation, SD: ±22) and 17 (SD: ±12) after vaccine introduction as summarized in Table 2. Hospital admissions per month in different hospitals ranged from 6 to 100.”  

**Comment**: It is likely to take time to build up vaccinated birth cohorts in the community, accordingly is it possible to assume that there is a “transition period”, and not only “before and after periods”?

**Response**: We agree with this and an analysis including two months wash out period has now been added as part of the sensitivity analysis.

**Comment**: Can the authors provide information on rotavirus vaccination coverage in Kenya over time?

**Response**: an extensive description has now been added.

**Comment**: The changes in DAD before and after introducing universal rotavirus vaccination might vary with age groups since the vaccine is given to infants up to 32 weeks of age. Thus, in the early period, the reduction might be of greater magnitude in infants than in toddlers. Therefore, it is important to explore the change separately for different age groups (e.g. 0-11, 12-23, and 24-36 months).

**Response**: We agree with you on this and an analysis exploring change for each age group has now been added as a sensitivity analysis.   Additional comments

**Comment**: Introduction – please provide up-to-date estimates (and references) of diarrheal disease burden.  

**Response**: This has now been added

**Comment**: Tables/ figures: there are typo errors. Legends should be added to explain the tables/abbreviations, and statistical analysis.

**Response**: This has been corrected and explanatory notes added.

**Comment**: Table 2: instead of “mean monthly admissions per hospital” please present the median and interquartile range.

**Response**: Both mean and median monthly admissions including the interquartile range have now been added

**Comment**: Table 3, under P value, instead of 0.00, please use <0.001.

**Response**: This has been corrected

**Comment**: In table 3 the authors presented odds ratios while in table 4 rate ratios. Please explain which models were used in each analysis.

 Table 4: please correct “ration” to ratios

**Response**: We regret this mix-up and has now been corrected to show rate ratios

**Comment**: Figure 3: there is a discrepancy in the title: above the figure, it is written “trends in surgical or burns admissions”, while the title underneath the figure is” Figure 3. Trends in hospitalization due to diarrhea and dehydration. Slope and level change in DAD hospitalizations over time”. Please check. In any case, this figure can be moved to supplementary material.

**Response**: We agree with you that there was a mix-up in naming and has been corrected.

**Comment**: In figures 2 and 3, please explain what the y-axis stands for rate or absolute numbers, as well as what the different colors of the dots represent

**Response**: We agree that this was not clear and has now been clarified

 References Ewusie, J. E., Soobiah, C., Blondal, E., Beyene, J., Thabane, L., & Hamid, J. S. (2020). Methods, applications and challenges in the analysis of interrupted time series data: a scoping review. *Journal of Multidisciplinary Healthcare*, 411-423. Guillaume, D. A., Justus, O. O., & Ephantus, K. W. (2020). Factors influencing diarrheal prevalence among children under five years in Mathare Informal Settlement, Nairobi, Kenya. *Journal of public health in Africa, 11*(1).

# Reviewer report: Reviewer response for version 1

**DOI:** 10.21956/wellcomeopenres.19260.r53844

**Published:** 2023-02-09

**Authors:** Dan Hungerford

**Affiliations:** 1 Institute of Infection, Veterinary and Ecological Sciences, University of Liverpool, Liverpool, UK

This study describes an ecological approach to assessing rotavirus vaccine impact on hospital admissions for diarrhoea with dehydration in Kenya across 13 hospitals. The analysis approach taken is an interrupted time-series analysis. 

 The study has major limitations.

 There is less than one pre-vaccine season included in the time-series analysis (10 months). For a time-series analysis to be robust for rotavirus there should be at least three seasons prior to vaccine introduction to allow for seasonal fluctuations.

 In the limitations the authors state that there are 10 data points which they specify is sufficient. But these are contained within one / potentially two rotavirus seasons. Timing of data points is crucial e.g. 10 data points representing days may be suitable for one intervention (e.g. in an acute outbreak of something with a short incubation period) whereas 10 years may be needed for another. This is even more important for a non-specific endpoint like DAD, where other pathogens may account for high numbers in the year preceding vaccine, were there any outbreaks of other enteric pathogens in 2013/14?

 Therefore, the authors should try and provide evidence from other studies in Kenya that yearly DAD admissions were at a consistent level prior to rotavirus vaccine introduction and that there were no enteric pathogen outbreaks or high seasons in the year prior to vaccine introduction.

 Another way to improve confidence in the findings would be to conduct an analysis of children 0-1 years of age as rotavirus vaccine impact would be expected to be greatest in this age group (see citation).

 Please could the authors also justify why an offset/denominator was not used in the model - using either total monthly admissions or catchment population size for the age group.

 There is no detail provide on population level vaccine uptake. This needs to be included in either the introduction as statements or ideally if data are available provided in the methods and results. If available there should also be regionally coverage figures provided as the study includes data from 13 hospitals.

 Other comments

 Abstract - Please add the study time period. Otherwise the abstract appears to be hiding the fact that there is limited pre-vaccine data.

 Introduction

 The reference for global diarrhoea cases is very old, either put in context that this is prior to rotavirus vaccine licensure or add a more recent estimate.

 Please also add detail that children experience many infections (symptomatic and asymptomatic) after first severe infection and immunity has variable waning by setting.

 Add some detail and references on the VE / impact of vaccination on rotavirus AGE in Kenya.

 Please also add a sentence or two on impact from other relevant countries - this provides useful context for the reader.

 Need to add context that the majority of severe infections will occur in children <2 years.

 Please add detail on which rotavirus vaccine is used in Kenya.

 Methods

 Please detail what coefficients / epi measures were generated from the models and how 95% CIs were generated.

 Results

 Table 3 presents odds ratios and table 4 rate rations? please clarify and as stated above specify transformation of coefficients in the methods.

Is the work clearly and accurately presented and does it cite the current literature?

Partly

If applicable, is the statistical analysis and its interpretation appropriate?

Partly

Are all the source data underlying the results available to ensure full reproducibility?

Yes

Is the study design appropriate and is the work technically sound?

Partly

Are the conclusions drawn adequately supported by the results?

Partly

Are sufficient details of methods and analysis provided to allow replication by others?

Partly

Reviewer Expertise:

Infectious disease epidemiology - focusing on GI. Specifically real world vaccine evaluations of rotavirus vaccines.

I confirm that I have read this submission and believe that I have an appropriate level of expertise to confirm that it is of an acceptable scientific standard, however I have significant reservations, as outlined above.

## References

[1] : Vaccines for preventing rotavirus diarrhoea: vaccines in use. *Cochrane Database Syst Rev* .2021;11(11) : 10.1002/14651858.CD008521.pub6 CD008521 10.1002/14651858.CD008521.pub6 34788488 PMC8597890

# Reviewer report: Reviewer response for version 1

**DOI:** 10.21956/wellcomeopenres.19260.r47771

**Published:** 2022-01-17

**Authors:** Mavuto Mukaka

**Affiliations:** 1 Mahidol Oxford Research Unit, Mahidol University, Bangkok, Thailand

The effect of introduction of routine immunization for rotavirus vaccine on paediatric admissions with diarrhoea and dehydration to Kenyan Hospitals: an interrupted time series study.

 The authors tackle an important area. However, there are a number of issues that must be addressed to improve the quality of this manuscript.

**Major:**

- In the abstract, the authors state that they used a segmented mixed effects model, but the statistical model used is not mentioned. They should state the statistical model.
- The authors state that they conducted multilevel multiple imputation to account for clustering of data within the hospitals. I thought the primary aim of multiple imputation is to address the missing data issue and not for accounting for clustering. Unfortunately, the authors do not state this primary aim of multiple imputation. It seems like there is a mix up of things. In that case which data was missing and was multiply imputed?
- For accounting for clustering, multilevel (hierarchical) models are relevant and authors need to state the type of statistical hierarchical model that was used and state the different levels of clustering.
- From the write-up, it is very difficult to capture the nature of the outcome. Table 3 provide odds ratios but logistic regression has never been mentioned anywhere in the text. Similarly, Table 4 provide rate ratios, are these incident rate ratios? Can the authors indicate in the table the model that was used to obtain these ratios? Was the outcome binary or count data?
- There is a mixed up between results and discussion. For example, in the results section about Changes in diarrhoea and dehydration after the introduction of rotavirus vaccine, the authors present the results in terms of percentage and 95% confidence intervals for the percentage change. However, the table being referred to presents odds ratios and 95% confidence intervals for the odds ratios. The authors should present the results as presented in the tables and they can make these other types of interpretations in the discussions. Presenting like this can easily confuse the readers when they crosscheck against the tables.
- Table 3, Level change is 1.25, 95% CI as 0.58 to 1.04. Why is the estimate 1.25 higher than the upper limit of the 95% CI i.e. 1.04?
- Figure 3 is a spaghetti plot of individual trajectiles, can the authors include the line that describes the overall trend i.e. the mean over time.
- Sensitivity analyses are described in the abstract and in the methods section but they seem not to be presented in the results section and discussed in the discussion section. The authors should present and discuss these.
- Authors should consider a brief section describing Missing at Random, Missing Not at Random and Missing completely at random definitions to help justify why Missing at Random was considered as a reasonable assumption.

**Minor:**

- Figure 1 says between “2013 and 2011”. It does not make sense to me. Please correct this. I do not see where 2011 is coming from.
- In table 4, the authors write “rate rations” instead of “rate ratios”.
- Table 2 misses some key variables including gender.
- P-values of 0 and 0.0 in the table are not meaningful. These p-values are conventionally presented as <0.001 etc. because the p-value cannot be exactly 0. It is also better to be consistent in the number of decimal places.

Is the work clearly and accurately presented and does it cite the current literature?

Partly

If applicable, is the statistical analysis and its interpretation appropriate?

Partly

Are all the source data underlying the results available to ensure full reproducibility?

No

Is the study design appropriate and is the work technically sound?

Yes

Are the conclusions drawn adequately supported by the results?

Yes

Are sufficient details of methods and analysis provided to allow replication by others?

Partly

Reviewer Expertise:

Statistics, epidemiology, malaria

I confirm that I have read this submission and believe that I have an appropriate level of expertise to confirm that it is of an acceptable scientific standard, however I have significant reservations, as outlined above.

# Response

**Published:** 2024-09-24

**Authors:** Daisy Chelangat

**Affiliations:** KEMRI-wellcome Trust, Kenya

**T** **he Effect of Introduction** **o** **f Routine Immunization** **f** **or Rotavirus Vaccine** **o** **n Paediatric Admissions** **w** **ith Diarrhoea** **a** **nd Dehydration** **t** **o Kenyan Hospitals: An Interrupted Time Series Study**

 Reviewer comments Major:

**Comment:** In the abstract, the authors state that they used a segmented mixed effects model, but the statistical model used is not mentioned. They should state the statistical model. **Response:** We have added the  model that was used in the analysis “To assess the impact of the rotavirus vaccine on DAD admissions, we applied a segmented mixed-effects negative binomial regression model”. Page 2

**Comment:** The authors state that they conducted multilevel multiple imputation to account for clustering of data within the hospitals. I thought the primary aim of multiple imputation is to address the missing data issue and not for accounting for clustering. Unfortunately, the authors do not state this primary aim of multiple imputation. It seems like there is a mix up of things. In that case which data was missing and was multiply imputed?

**Response:** We agree with the reviewer that the aim of multiple imputation was not clear. We have clarified this and also given the justification for multilevel multiple imputation. “We then performed  multilevel multiple imputation to impute missing data, accounting for potential clustering of missingness within the hospitals”. Page 6  

**Comment:** For accounting for clustering, multilevel (hierarchical) models are relevant, and authors need to state the type of statistical hierarchical model that was used and state the different levels of clustering.

**Response: **This has been corrected in the manuscript “fitting a segmented mixed-effects negative binomial regression model with an autoregressive covariance structure and a log link function to examine step and slope changes in DAD admission counts”. Page 6

**Comment:** From the write-up, it is very difficult to capture the nature of the outcome. Table 3 provide odds ratios but logistic regression has never been mentioned anywhere in the text. Similarly, Table 4 provide rate ratios, are these incident rate ratios? Can the authors indicate in the table the model that was used to obtain these ratios? Was the outcome binary or count data?

**Response:** The outcome variable has now been clarified “In this study, we use routinely collected data and an interrupted time series design to assess changes in all-cause severe diarrhoea admissions following the introduction of the vaccine,”. Page 3. It has been clarified in the tables that we are presenting results from an “Interrupted time series analysis coefficients for diarrhea and dehydration admissions” (Table 2). For the odds ratios, a negative binomial mixed effect regression model with a log odds link function gives odds ratio estimates for coefficients.  

**Comment:** There is a mixed up between results and discussion. For example, in the results section about Changes in diarrhoea and dehydration after the introduction of rotavirus vaccine, the authors present the results in terms of percentage and 95% confidence intervals for the percentage change. However, the table being referred to presents odds ratios and 95% confidence intervals for the odds ratios. The authors should present the results as presented in the tables and they can make these other types of interpretations in the discussions. Presenting like this can easily confuse the readers when they crosscheck against the tables. **Response:** We agree that there is a mix up and the discussion section has been re-written for clarity. Page 12

**Comment:** Table 3, Level change is 1.25, 95% CI as 0.58 to 1.04. Why is the estimate 1.25 higher than the upper limit of the 95% CI i.e. 1.04?

**Response:** This was a typing error from the previous section and has now been corrected      and is in Table 3; Level change is 1.25 and the 95% CI is 0.58 to 1.54

**Comment:** Figure 3 is a spaghetti plot of individual trajectiles, can the authors include the line that describes the overall trend i.e. the mean over time.

**Response:** A graph that now describes overall trend along side the individual trajectories has now been added in the same panel (Figure2 and 3).

**Comment:** Sensitivity analyses are described in the abstract and in the methods section but they seem not to be presented in the results section and discussed in the discussion section. The authors should present and discuss these.

**Response:** In the results and discussion sections, the sensitivity analysis is presented but not clearly. An extensive description of a couple of sensitivity analyses is now presented.  

**Comment:** Authors should consider a brief section describing Missing at Random, Missing Not at Random and Missing completely at random definitions to help justify why Missing at Random was considered as a reasonable assumption.

**Response:** A section describing different mechanisms of missing data has now been added as a supplementary material  

**Minor:**

**Comment:** Figure 1 says between “2013 and 2011”. It does not make sense to me. Please correct this. I do not see where 2011 is coming from.

**Response:** this was a typing error and has been corrected in Figure 1 which now reads: “September 2013 to November 2021” 

**Comment:** In table 4, the authors write “rate rations” instead of “rate ratios”.

**Response:** This was a typing error and it now reads: “rate ratios”. Table 2

**Comment:** Table 2 misses some key variables including gender.

**Response:** Variable gender has now been added and is now presented in Table 1

**Comment:** P-values of 0 and 0.0 in the table are not meaningful. These p-values are conventionally presented as <0.001 etc. because the p-value cannot be exactly 0. It is also better to be consistent in the number of decimal places.

**Response:** This is now corrected, and p values now reads “<0.01”. The number of decimal places is now also consistent across the manuscript.
